# Influence of hunting strategy on foraging efficiency in Galapagos sea lions

**DOI:** 10.7717/peerj.11206

**Published:** 2021-04-13

**Authors:** Jessica-Anne Blakeway, John P.Y. Arnould, Andrew J. Hoskins, Patricia Martin-Cabrera, Grace J. Sutton, Luis A. Huckstadt, Daniel P. Costa, Diego Páez-Rosas, Stella Villegas-Amtmann

**Affiliations:** 1 School of Life and Environmental Sciences, Deakin University, Burwood, VIC, Australia; 2 CSIRO Health and Biosecurity, Townsville, Queensland, Australia; 3 Department of Ecology & Evolutionary Biology, University of California, Santa Cruz, CA, United States of America; 4 Department of Ecology & Evolutionary Biology, University of California, Santa Cruz, United States of America; 5 Universidad San Francisco de Quito and Galapagos Science Center, Islas Galápagos, Ecuador; 6 Dirección Parque Nacional Galápagos, Oficina Técnica Operativa San Cristóbal, Islas Galápagos, Ecuador

**Keywords:** Telemetry, Pinniped, *Zalophus wollebaeki*, Dive behaviour, Galapagos Islands, Accelerometers, Feeding

## Abstract

The endangered Galapagos sea lion (GSL, *Zalophus wollebaeki*) exhibits a range of foraging strategies utilising various dive types including benthic, epipelagic and mesopelagic dives. In the present study, potential prey captures (PPC), prey energy consumption and energy expenditure in lactating adult female GSLs (*n* = 9) were examined to determine their foraging efficiency relative to the foraging strategy used. Individuals displayed four dive types: (a) epipelagic (<100 m; EP); or (b) mesopelagic (>100 m; MP) with a characteristic V-shape or U-shape diving profile; and (c) shallow benthic (<100 m; SB) or (d) deep benthic (>100 m; DB) with square or flat-bottom dive profiles. These dive types varied in the number of PPC, assumed prey types, and the energy expended. Prey items and their energetic value were assumed from previous GSL diet studies in combination with common habitat and depth ranges of the prey. In comparison to pelagic dives occurring at similar depths, when diving benthically, GSLs had both higher prey energy consumption and foraging energy expenditure whereas PPC rate was lower. Foraging efficiency varied across dive types, with benthic dives being more profitable than pelagic dives. Three foraging trip strategies were identified and varied relative to prey energy consumed, energy expended, and dive behaviour. Foraging efficiency did not significantly vary among the foraging trip strategies suggesting that, while individuals may diverge into different foraging habitats, they are optimal within them. These findings indicate that these three strategies will have different sensitivities to habitat-specific fluctuations due to environmental change.

## Introduction

The ability to forage successfully and efficiently is crucial to the survival and the overall fitness of animals (Pyke, 1984). Foraging behaviour has been linked to factors such as age, experience, competition, and reproductive status (Hoskins et al., 2015; Sutton et al., 2020). To efficiently forage, individuals must also adapt their foraging behaviour to their environment. Correspondingly, numerous studies have revealed variation in trophic niche and foraging behaviour exists within and between populations in relation to environmental conditions (Bolnick et al., 2003). As foraging efficiency can strongly impact reproductive success, population growth and, in turn, the survival of a species (Pyke, 1984), knowledge of the factors influencing it is important for understanding the impacts of environmental change on species (Gallagher et al., 2015).

In air-breathing marine predators such as seabirds, cetaceans (whales and dolphins) and pinnipeds (seals, sea lions and walruses), foraging is constrained by the need to return to the surface for oxygen (Butler & Jones, 1997; Andrews & Enstipp, 2016). Two main foraging strategies have evolved (pelagic and benthic), with the main variations being time spent at the bottom phase of the dive (Tremblay & Cherel, 2000; Arnould & Costa, 2006) and the dispersion of prey items (Chilvers & Wilkinson, 2009; Doniol-Valcroze et al., 2011). Pelagic foragers exhibit “V-Shaped” dives that exploit prey throughout the water column with little time at the bottom phase of the dive whereas benthic dives (flat bottom dives) are characteristic of animals that hunt along the seafloor for benthic or demersal prey. While benthic prey are found in lower densities and are often solitary, they tend to be more predictable and evenly dispersed then pelagic prey that tend to be found in spatiotemporally unpredictable, high-density patches (Tremblay & Cherel, 2000; Chilvers & Wilkinson, 2009; Doniol-Valcroze et al., 2011).

Pinnipeds show high heterogeneity in foraging behaviour linked to habitat productivity and morphometrics (Páez-Rosas, Villegas-Amtmann & Costa, 2017). In addition, pinnipeds exhibit energetically expensive lifestyles due to the thermoregulatory costs of the aquatic environments in which they acquire food (Williams et al., 2000). Foraging strategies are known to vary not only between populations but also within the same demographic groups (Hoskins et al., 2015; Volpov et al., 2015). As top predators, pinnipeds can adapt to a wide range of foraging strategies, enabling them to better adjust to competition pressures, energy investments and resource variation (Chilvers & Wilkinson, 2009; McHuron et al., 2016). Although many studies have investigated diet, niche separation and habitat preferences in marine predators, little is known how various foraging strategies influence foraging efficiency.

The Galapagos sea lion (GSL, *Zalophus wollebaeki*) is the only sea lion to have adapted to tropical environment of the equatorial Pacific Ocean (Churchill, Boessenecker & Clementz, 2014). It is endemic to the Galapagos Islands, a region where levels of marine productivity are strongly influenced by seasonal oceanographic currents and a pattern of upwelling that make this area a biodiversity hotspot (Palacios et al., 2006; Schaeffer et al., 2008). The GSL are found on most islands within the archipelago and are central place foragers throughout the protracted lactation period, during which pups are dependent on their mothers (Trillmich, 1986; Trillmich & Wolf, 2008). As adult females need to regularly return to the colony to feed their offspring, efficient foraging strategies are paramount for their success (Villegas-Amtmann & Costa, 2017).

The GSL population has declined approximately 50% over the last 40 years (Trillmich, 2015). It has been suggested that this is due in part to the increasing frequency of extreme El Niño-Southern Oscillation (ENSO) events which impact resource predictability, leading to reduced pup size and increased pup mortality for GSL (Trillmich & Dellinger, 1991; Trillmich, 2015). The small geographical range of the GSL provides potential complications, as individuals cannot migrate to different regions to evade lower biological productivity associated with the increasing warming waters or to exploit different food resources in response to fluctuations in the environment (Schaeffer et al., 2008; Cai et al., 2014).

Previous studies have documented a variety of foraging strategies with the GSL population, reflecting the diversity of habitats available within the Galapagos Archipelago (Villegas-Amtmann et al., 2008; Páez-Rosas & Aurioles-Gamboa, 2010). These strategies have been linked to factors such as age (Jeglinski et al., 2012), colony (Páez-Rosas & Aurioles-Gamboa, 2014), and prey availability (Dellinger & Trillmich, 1999; Páez-Rosas, Villegas-Amtmann & Costa, 2017). However, the relative benefits of the different foraging strategies are unknown (Trillmich et al., 2014). Knowledge of difference in foraging efficiency associated with different strategies may provide insights into the impacts of future changes to the Galapagos marine ecosystem (Trillmich et al., 2014). The aims of this study, therefore, were to determine: (1) prey capture rates and energy consumption; (2) energy expenditure and foraging efficiency; and (3) the influence of dive type and foraging trip strategy on foraging efficiency in lactating adult female GSLs.

## Materials and Methods

### Study site and data collection

This study was undertaken as part of the GSL population-monitoring program conducted by the Galapagos National Park Directorate (GNPD) and the Universidad San Francisco de Quito (USFQ) under research permits PC-19-12, and PC-61-13. The methods described here were reviewed and approved by the USFQ and GNPD committee responsible for assessing animal welfare in research activities.

**Figure 1:**
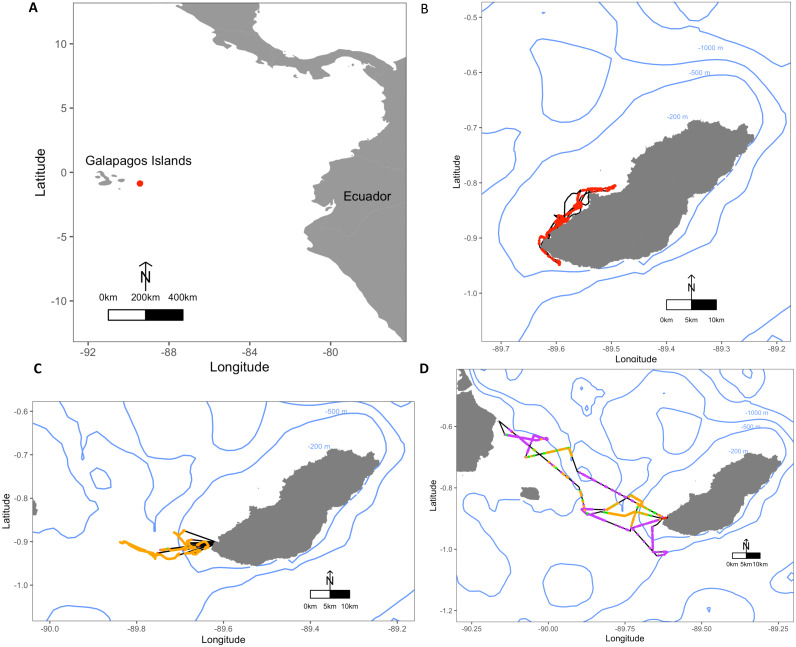
Maps depicting the study site El Malecon Colony in November 2012 and representative trips of various Foraging Trip Types utilised by individuals. (A) The Galapagos Islands relative to the South American coastline and Ecuador with the study site at the El Malecon Colony represented by a red dot. Galapagos sea lion GPS tracks leaving from the colony representative of (B) Group 1, (C) Group 2 and (D) Group 3. Potential Prey Captures (PPC) within trips are coded to represent dive strategy utilized overlaid on the bathymetry highlighted by blue contours (200 m, 500 m and 1,000 m). The track = black line, Shallow Benthic (SB) = red, Deep Benthic (DB) = purple, Epipelagic (EP) = orange, Mesopelagic (MP) = green.

This study was conducted at the El Malecon colony, San Cristóbal Island, Galapagos Islands (0.9019°S, 89.6142°W; Fig. 1) from November–December 2012 (i.e., during a La Niña phase (NOAA, 2020)). The El Malecon colony is the largest colony of GSL and San Cristóbal Island hosts approximately 13% of the GSL population (Riofrío-Lazo, Arreguín-Sánchez & Páez-Rosas, 2017). Adult females nursing pups 1–3 months old (Riofrío-Lazo, Arreguín-Sánchez & Páez-Rosas, 2017) were selected opportunistically according to ease of capture access at the site. Individuals were captured using a modified hoop net before being tranquillised with a 0.8-1 mL intra-muscular injection of Telazol (teletamine/zolazepam HCl) at a 100mg/ml concentration. Once tranquillised, individuals were weighed using a sling, tripod and a digital scale (±0.1 kg). Standard length and axillary girth (Mammals, 1967) were recorded using a tape measure (±0.5 cm).

Individuals were instrumented with an ARGOS satellite telemetry transmitter or Fastloc GPS data logger combined with a dive behaviour data logger (MK9-AF and MK10-AF, Wildlife Computers, Richmond, WA, USA). The GPS devices were programmed to record spatial locations every 15 min with an estimated accuracy of 0.5–11 km (Costa et al., 2010). Dive behavior loggers were set to sample depth (± 0.5 m) at 2 s intervals. Instruments were attached to a neoprene layer and mesh netting and glued with 5-min epoxy (Loctite) to the dorsal midline between the scapula. A VHF radio transmitter (Sirtrack, Havelock North, New Zealand) was also attached to the dorsal midline posterior to the data loggers to assist in relocation for recapture. A tri-axial accelerometer data logger (X6, Gulf Coast Data Concepts, Waveland, MS, USA) was attached using the same method to the top of the head between the ears and programmed to sample at 25 Hz. Animal handling lasted <30 min before individuals were released to resume normal behaviours. Individuals were recaptured after 1–7 foraging trips to sea and the devices removed by cutting through the neoprene layer underneath them. The residual epoxy mounts fall off within a few months during the animals’ annual moult.

### Dive behaviour and prey encounter events

Dive behaviour data were initially zero-offset corrected (ZOC) to account for pressure sensor drift and summary statistics for each dive were extracted using the *diveMove* package (Luque, 2007) within R statistical environment version 3.5.2 (R Core Team, 2018). Only submergences exceeding a depth >5 m and durations >12 s were considered foraging dives and included in analyses (Jeglinski et al., 2012; Villegas-Amtmann et al., 2013). The remaining 10261 foraging dives were visually classified based on depth and dive profile as: (a) epipelagic (<100 m; EP); or (b) mesopelagic (>100 m; MP) if with V-shaped or U-shaped profiles; and (c) shallow benthic (<100 m; SB) or (d) deep benthic (>100 m; DB) if with square or flat-bottom dives.

The GPS positions were decoded and filtered using a speed filter of 6 km h^−1^ to remove erroneous locations (Villegas-Amtmann et al., 2013). Satellite data (ARGOS) were pre-processed using a conservative forward/backward speed filter of 10 m s^−1^ to remove erroneous locations (Patterson et al., 2010). The ARGOS data were additionally processed using a State Space Model (SSM) in order to obtain positions estimates that included errors related to each ARGOS location class and the dynamics of the movement process (Jonsen, Flemming & Myers, 2005; McConnell et al., 2010; Patterson et al., 2010). Both the GPS and ARGOS location data were then interpolated at 10-s intervals and merged with dive summaries. Female GSL are known to haul-out in multiple locations within a foraging trip (Villegas-Amtmann et al., 2008; Montero-Serra et al., 2014). Consequently, at-sea tracks were visually analysed to determine when an individual left and returned to the colony to define the start and end times of a foraging trip in the dive record. In addition, periods when individuals were hauled-out were subtracted from the duration away from the natal colony to obtain the effective time at sea. As GSL enter the water near the colony to thermoregulate, only departures from the colony >2 h were considered foraging trips.

The diving data from the individual foraging trips were merged with the tri-axial accelerometer data in the software package IgorPro (WaveMetrics, Lake Oswego, OR, USA). Potential prey captures (PPC) were estimated from peaks in the *Y*-axis (sway) acceleration data using the *Ethographer* package (Sakamoto et al., 2009) in IgorPro following the methods of Volpov et al. (2015) and Foo et al. (2016). Briefly, a 3-Hz high-pass filter was applied to the sway acceleration to isolate head movements from movements associated with the body (Viviant et al., 2010). A 1.5-s moving time window was then applied to the filtered data to determine the standard deviation (SD) and a threshold was calculated above which peaks were classified as a PPC. As peaks occurring within quick succession could reflect prey handling rather than additional prey encounters, a minimum time interval was used between two successive peaks to separate PPC. This method of identifying PPC has been validated using animal-borne video cameras on free-ranging female Australian Fur Seals (*Arctocephalus pusillus doriferus*) (Volpov et al., 2015).

To identify the optimal threshold on the SD of the sway acceleration and minimum time interval for detecting PPC in GSL, a range of threshold values from 0.1 to 0.6 g were tested for each individual. Thresholds were determined by: (1) assessing the dominant SD of the sway acceleration values within a frequency histogram; and (2) examining the raw acceleration data (Foo et al., 2016). The threshold was then chosen based on the highest rate of change. Minimum time intervals increasing by 0.5 s from 1–7 s were used to calculate PPC and the rate of change in PPC detection for each individual. The optimal minimum time interval was selected using the same methods used for the optimal threshold. A threshold on the SD of the sway acceleration of 0.4 and minimum time interval of 4 s was selected to determine PPCs for all GSL individuals except for one. For this individual the SD of a sway acceleration threshold of 0.2 and a minimum time interval of 5 s were selected due to a reduced detection rate. This was likely due to the placement of the accelerometer causing inaccurate sampling (Preston, Baltzer & Trost, 2012).

### Energy expenditure and foraging efficiency

Previous studies have shown a correlation between vectorial dynamic body acceleration (VeDBA), calculated from the tri-axial accelerometer data, and energy expenditure in a range of species (Qasem et al., 2012; Jeanniard-du Dot et al., 2017a; Grémillet et al., 2018; Sutton et al., 2020). Correspondingly, static acceleration (S; due to individual’s position relative to gravity) was calculated for each axis (*x*, *y* and *z*) using a 2-s running mean and subtracted from the acceleration (A) to provide dynamic acceleration due to movement (Shepard et al., 2008; Qasem et al., 2012). The VeDBA throughout complete foraging trips was calculated using the equation:

\begin{eqnarray*}VeDBA=\sqrt{({A}_{x}-{S}_{x})^{2}+({A}_{y}-{S}_{y})^{2}+({A}_{z}-{S}_{z})^{2}}. \end{eqnarray*}

The resulting VeDBA values were used to estimate energy expenditure (MJ kg^−1^) from conversion equations previously determined in the Antarctic (*Arctocephalus gazella*) and northern (*Callorhinus ursinus*) fur seals (Jeanniard-du Dot et al., 2017a) during diving and while at the surface:

\begin{eqnarray*}& & E{E}_{\text{dive}} \left( \text{MJ}{\text{kg}}^{-1} \right) \sim \left( 0.10\pm 0.10 \right) + \left( 91.99\pm 4.42 \right) \times {VeDBA}_{\text{dive}}({\text{ms}}^{-2}{\text{kg}}^{-1}\ast \text{day}) \end{eqnarray*}

\begin{eqnarray*}& & E{E}_{\text{surface}} \left( \text{MJ}{\text{kg}}^{-1} \right) \sim \left( 0.06\pm 0.07 \right) + \left( 23.40\pm 1.48 \right) \times {VeDBA}_{\text{surface}}({\text{ms}}^{-2}{\text{kg}}^{-1}\ast \text{day}) \end{eqnarray*}

\begin{eqnarray*}& & \text{Total}~EE\sim E{E}_{\text{dive}}+E{E}_{\text{surface}}. \end{eqnarray*}

Estimates of the energy content (EC) gained by PPC were obtained from information on the prey items (species and size) reported in a concurrent diet study on GSL at the same location (Páez-Rosas, Villegas-Amtmann & Costa, 2017) and energy content values for these species derived from the literature (Table S1). Prey species were classified according to their habitat to obtain an average prey energy content for the different dive types identified (Lee, Gelwick & Davis, 2010). Estimated EC gained by individual GSL was then calculated based on the PPC and the dive type in which they occurred.

Foraging efficiency (FE; kJ s^−1^) was calculated from the estimated gross energy expenditure and the total energy gained while foraging using the equation:

\begin{eqnarray*}\mathrm{FE}= \frac{{\mathrm{GE}}_{\text{consumed}}-G{E}_{\text{expended}}}{{\text{time}}_{ \left( \text{s} \right) }} \end{eqnarray*}

where GE_consumed_ is the number of prey items multiplied by the estimated EC of potential prey items based on Dive Type for individuals’ dives or summed across a foraging trip, and GE_expended_ is the energy expended during individual dives or the entire foraging trip.

Linear mixed-effects models (LMEs) were used to evaluate the influence of Dive Type on the response variables of PPC, energy consumed, energy expended and foraging efficiency estimates using the *lme4* (Bates et al., 2015) package in R. The individual was categorised as a random factor to account for repeated measurements (dives) within individuals. Parameters describing individual components of dives such as dive depth, duration, and bottom time were not considered part of the modelling process due to their use, and thus collinearity, in the classification of dive types. Model residuals were tested for skewness by visually assessing boxplots, and no transformations were necessary. Differences between Dive Types were evaluated using an ANOVA run on the model outputs, which were followed by Tukey’s multiple comparisons using the *multcomp* package (Hothorn, Bretz & Westfall, 2008).

As individuals displayed multiple Dive Types within and between foraging trips, to investigate factors influencing potential prey capture rates and FE at the trip level a multivariate hierarchical classification analysis was conducted. The classification using Euclidean distance and the ward D2 linking method (Murtagh & Legendre, 2014) within the *pvclust* package (Suzuki & Shimodaira, 2006) and groupings were based on a 95% confidence interval. Dive parameters used for cluster analysis included: total time at sea (h); total number of haul-outs; total time at haul-out (h); vertical dive rate (m s^−1^); percentage of trip spent diving (%); percentage of dives using DB, SB, EP or MP Dive Types; mean bottom time (s); mean dive duration (s); mean maximum depth (m); mean post-dive duration (s); and ascent and descent rates (m s^−1^). Where means were used, standard deviations and skewness values were included to account for variation within the data. The resultant groupings were considered as Foraging Trip Types.

To assess the influence of Foraging Trip Type on PPC, gross energy consumed and expended, and foraging efficiency, LMEs were created with Foraging Trip Type as a fixed effect and individual as a random effect. LMEs were analysed using the same process outlined above. Parameters describing individual components of Foraging Trip Types that were used in cluster analysis were not considered part of the modelling process due to their use, and thus collinearity, in the classification of Foraging Trip Types. As morphological features have been linked to foraging behaviours in GSL (Jeglinski et al., 2013), their influence on Foraging Trip Type was investigated with linear mixed effect models (Bates et al., 2015). To avoid pseudo-replication issues, these analyses were not conducted at the Dive Type level. Other factors such as pup size and sex were not included in analysis as the data were not available.

All statistical analyses were conducted in the R statistical environment (version 1.1.463). Unless otherwise specified, all further data are reported as mean ± SE, and results considered significant at *P* < 0.05. Bathymetry for maps were reproduced from the GEBCO Compilation Group (2019) website (https://www.gebco.net/) and land data were sourced from the CIAT-CSI SRTM website (http://srtm.csi.cgiar.org).

## Results

Dive behaviour with matched accelerometer data were obtained from 9 individuals (*n* = 9), over 7.4 ± 0.5 days for each individual and a range of 4.4 to 9.6 days. Spatial data were only available for 8 animals due to device failure. Individuals weighed 67.5 ± 1.9 kg, with standard lengths of 151.3 ± 1.5 cm and mean axillary girths of 96.1 ± 1.4 cm (Table 1).

**Table 1:** Summary of deployment and morphometric information for Galapagos sea lions from the El Malecon Colony in November 2012.

ID	Deployment date	Duration (d)	Mass (kg)	STDL (cm)	BCI_1_ (kg/cm)	Axillary girth (cm)	BCI_2_
GSL1	20/11/12	8.1	72	160	0.45	101	0.631
GSL2	20/11/12	6.7	57.2	152	0.375	90	0.592
GSL3	20/11/12	4.4	68.4	150	0.456	100	0.667
GSL5	21/11/12	6.3	71.2	149	0.478	94	0.631
GSL6	21/11/12	8.4	66.8	147	0.454	97	0.660
GSL7	22/11/12	9.6	68	151	0.45	99	0.656
GSL8	22/11/12	7.3	62.2	154	0.404	89	0.578
GSL9	22/11/12	8.3	64.8	145	0.447	98	0.676
GSL10	22/11/12	7.7	76.6	154	0.497	97	0.630
Mean ± SE		7.4 ± 0.5	67.5 ± 1.9	151.3 ± 1.5	0.446 ± 0.012	96.1 ± 1.4	0.636 ± 0.011

**Notes.**

Data include: Date of deployment, duration of data collection in days (Duration), body mass, standard length (STDL), body condition index (BCI1 = mass/STDL; BCI2 = axillary girth/STDL), and axillary girth were recorded prior to deployment. Group means ( ± S.E.) for all of the metrics are also provided.

Average foraging trips were 31.8 ± 5.3 h in duration, completing 282.7 ± 32.6 dives during 28.6 ± 4.5 h at sea. At sea, 47.9 ± 2.4% of time was spent diving. Dive depths varied greatly among individuals, with a maximum recorded depth of 568.5 m, and mean dive depths ranging between 20.7 ± 6.4 m and 163.5 ± 166.1 m. A total of 10,261 foraging dives were recorded with 1,140.1 ± 139.9 recorded per individual ranging between 541 and 1,821 dives. Dive Types and dive parameters varied both among individuals and within individuals (Table 2). Some individuals consistently stayed close to San Cristobal, often foraging within the 200 m bathymetry contour, whereas other individuals utilised a range of depths and travelled to different islands (Fig. 1).

**Table 2:** Trip and dive summary (Mean ± S.E.) of Galapagos sea lions from the El Malecon Colony in November 2012.

ID	Trip (*n*)	Trip duration (h)	Time at sea (h)	Dives (*n*)	Dive depth (m)	Max depth (m)	Dive duration (min)	Dive type (%)				PPC (*n*)	PPC rate (PPC^−^ dive^−1^)
								**SB**	**DB**	**EP**	**MP**		
GSL1	5	22.9 ± 7.6	19.2 ± 5.6	1,172	27.3 ± 12.6	87	2.4 ± 0.0	100				920	0.9 ± 0.0
GSL2	6	16 ± 1.6	15.2 ± 1.2	1,260	33.9 ± 16.8	102	2.4 ± 0.0	97.2	0.1	2.7		438	0.4 ± 0.0
GSL3	1^p^	105.7	86.6	859	46.9 ± 59.5	315	1.8 ± 0.1	0.9	11.5	86.6	0.9	539	0.6 ± 0.0
GSL5	2	62.9 ± 7.9	62.9 ± 7.9	878	125.7 ± 156.2	568.5	3.8 ± 0.1	4.6	20.2	61.1	14.2	1,917	2.2 ± 0.1
GSL6	2	81.3 ± 10.3	61.4 ± 13	1,267	81.5 ± 90.6	470	2.6 ± 0.1	3.3	20.5	68.7	7.5	1,399	1.1 ± 0.1
GSL7	1	86.4	71.4	789	163.5 ± 166.1	476.5	4.5 ± 0.1	0.8	37.3	54.0	8.0	2,055	2.6 ± 0.1
GSL8^*^	–	–	–	1,821	20.7 ± 6.4	81.5	2.1 ± 0.0	99.6		0.4		452	0.3 ± 0.0
GSL9	2	48.9 ± 18.2	48.9 ± 18.2	541	160 ± 89	431.5	5.4 ± 0.1	4.3	68.9	11.7	15.2	1,490	2.8 ± 0.1
GSL10	7	15.8 ± 3.8	15.8 ± 3.8	1,674	29.5 ± 24.5	264	1.5 ± 0.0	2.6	1.3	95.5	0.6	712	0.4 ± 0.2

**Notes.**

Data include: trip duration, time at sea, dive depth, maximum depth, dive duration, total number of complete trips (Trips) and recorded dives (Dives), percentage of dives using shallow benthic (SB), deep benthic (DB), epipelagic (EP) or mesopelagic (MP) dive types, total number of potential prey captures per individual (PPC) and rate of PPC per dive (PPC rate).

P Only partial dive log and accelerometer data available for trips.

* There was no spatial data available such that it was not possible to allocate dive to specific foraging trip.

**Figure 2:**
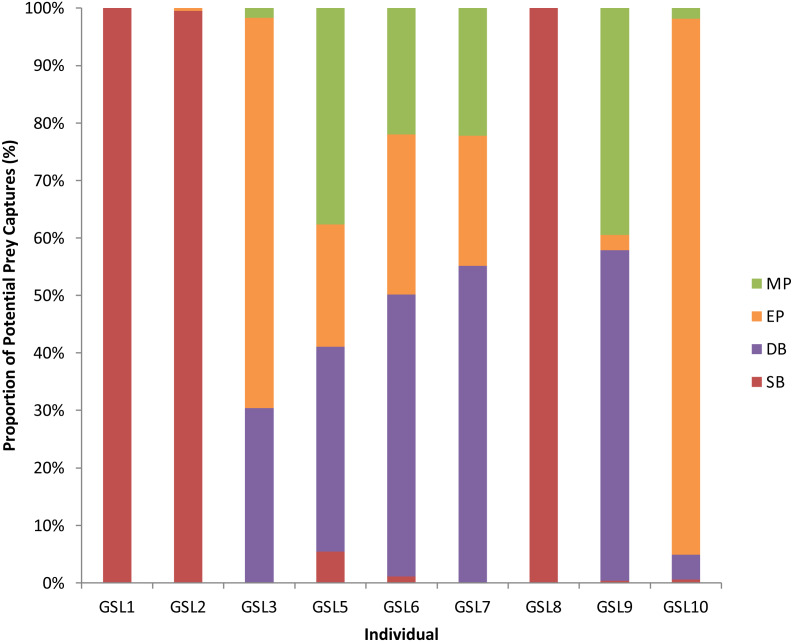
Potential Prey Captures (PPC) recorded for individual Galapagos sea lions from the El Malecon Colony as recorded in November 2012 (*n* = 9). Bar graph displaying the proportion of PPC occurring during the following Dive Types: Shallow Benthic (SB) = red, Deep Benthic (DB) = purple, Epipelagic (EP) = orange and Mesopelagic (MP) = green.

A total of 3,851 dives (35.5% of the total) had PPCs, resulting in a total of 9,922 PPC and a rate of 2.6 PPC per successful dive. The LME results indicated an influence of Dive Type on the number of PPC, with the ANOVA on the model showing significant differences between Dive Types (*F*_3_ = 1, 111.36, *P* < 0.0001: Table S2). Tukey’s *post-hoc* analysis revealed significant differences between all Dive Types (*P* < 0.0001 in all cases: Table S3) except between Epipelagic (EP) and Shallow Benthic (SB) (*P* = 0.336). The SB and EP dives were the most common, with similar PPC rates (0.4 ± 0.0 and 0.5 ± 0.0, respectively). The Mesopelagic (MP) dives were less common, making up 0.04% of dives but proportionately had the highest PPC rate per dive (5.3 ± 0.2). The PPC rate during Deep Benthic (DB) dives (2.86 ± 0.07) was approximately half of the MP PPC rate (Table 3). The Dive Type utilised by individuals varied with some individuals only having PPC within one Dive Type, where others completed PPC within a range of Dive Types (Fig. 2). Individuals that completed PPC in a range of Dive Types had a higher overall PPC rate and a greater variety of targeted prey items (Table S1).

**Table 3:** Summary table of the number of dives, number of Potential Prey Captures (PPC) and prey capture rates recorded in Galapagos sea lions from the El Malecon Colony in November 2012.

Dive type	PPC (*n*)	Dives (*n*)	PPC rate (dive^−1^)
SB	1,937	4,373	0.4 ± 0.0
DB	3,555	1,241	2.9 ± 0.1
EP	2,334	4,252	0.5 ± 0.0
MP	2,096	395	5.3 ± 0.2
Total	9,922	10,261	1.0 ± 0.0

**Notes.**

Data are presented as Mean ± SE for each dive type: shallow benthic (SB), deep benthic (DB), epipelagic (EP), and mesopelagic (MP) dive types.

**Figure 3:**
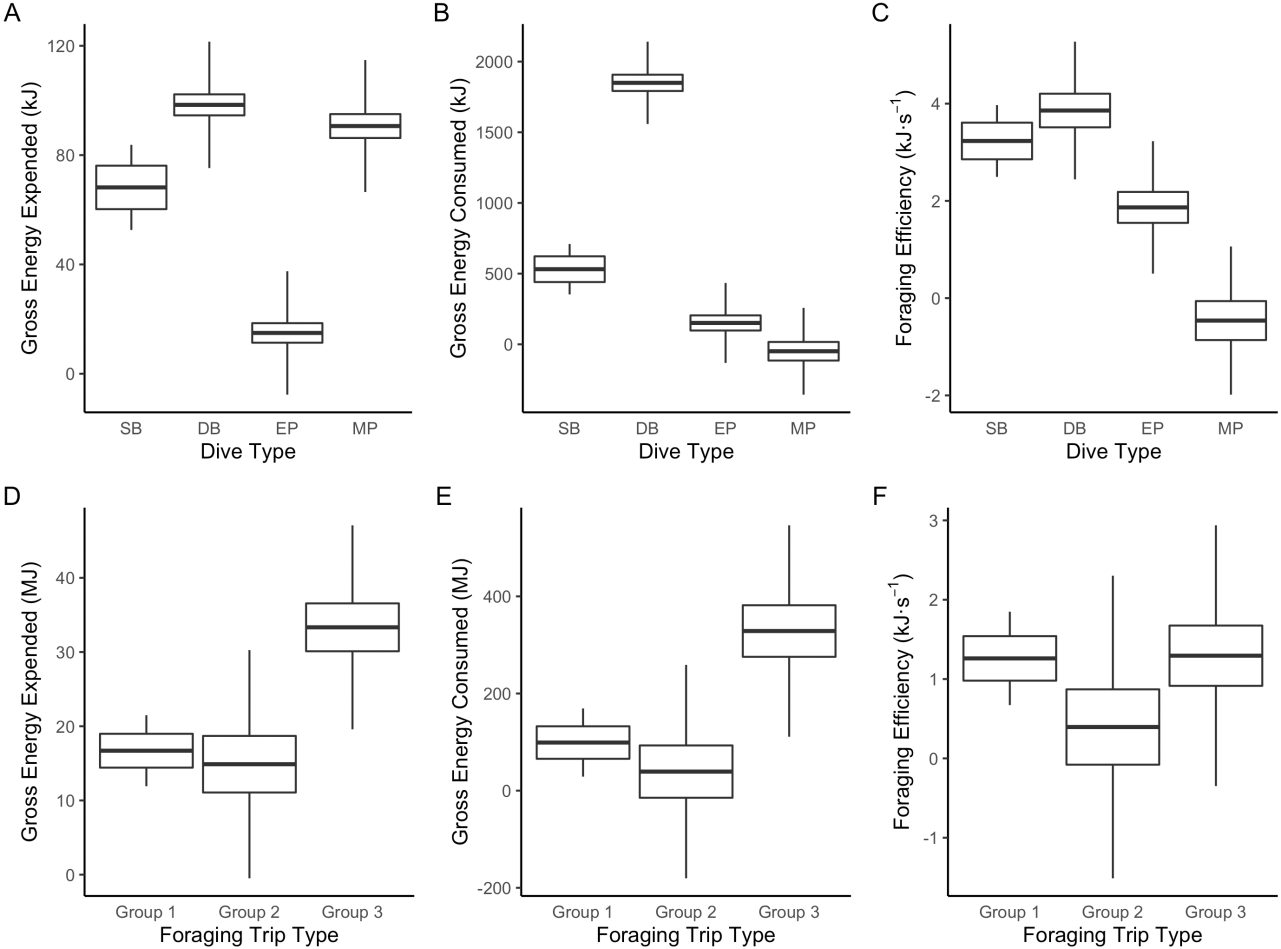
Boxplot comparisons of estimated energy expended, energy consumed and foraging efficiency between Dive Types and Foraging Trip Types in Galapagos sea lions from the El Malecon Colony in November 2012. (A) Gross energy expended (kJ), (B) gross energy consumed (kJ) and (C) foraging efficiency (kJ s^−1^) per Dive Type (SB, shallow benthic; DB, deep benthic; EP, epipelagic and MP, mesopelagic) and (D) gross energy expended (MJ), (E) gross energy consumed (MJ), and (F) foraging efficiency (kJ s^−1^) per Foraging Trip Types (Group 1 = GSL 1 and GSL 2, Group 2 = GSL 10 and Group 3 = GSL 5, 6, 7 and 9).

The LME results indicated that Dive Type had a strong influence on the energetic consequences of the dives. The ANOVAs on the model outputs revealed significant differences between Dive Types for energy consumed (*F*_3_ = 1,276.6, *P* < 0.001), energy expended (*F*_3_ = 754, *P* < 0.0001) and foraging efficiency estimates (*F*_3_ = 87.6, *P* < 0.0001). Tukey’s *post-hoc* comparisons revealed all Dive Types were significantly different (*P* < 0.05 in all cases) from each other for all response variables except SB and DB in foraging efficiency (*P* > 0.2). The DB dives were associated with the greatest amounts of prey energy consumed but also energy expended. While MP and SB dives had the lowest prey energy consumed, SB had low levels of energy expenditure whereas MP had proportionally high-energy expenditure (Fig. 3).

The hierarchical clustering analysis established three distinct Foraging Trip Types (Fig. S1). Group 1 consisted of all trips from individuals GSL1 and GSL2, which were predominately characterised by SB dives (98.5 ± 0.6%). Of the three Trip Types, Group 1 was the only one not to utilise all dives types (no MP). Individuals from this group spent approximately 17 ± 2.5 h at sea with 2.1 ± 1.2 h spent hauled out at sites other than the colony, and dived to maximum depths of 33.5 ± 3.9 m for 2.6 ± 0.2 min. In general, Group 1 spent the greatest proportion of time at sea diving (54.8 ± 3.9%) and completed 219 ± 39 dives per trip. Group 2 was comprised of trips completed solely by GSL10 which primarily conducted EP dives (95.3 ± 1.8%), spending 15.8 ± 3.8 h at sea, with no haul-outs recorded away from the colony and completed an average of 237 ± 40 dives per trip. Similar to Group 1, GSL10 dived to maximum depths of 29.4 ± 2.8 m, although dive durations were shorter (1.5 ± 0.1 min). Groups 1 and 2 were similar, foraging closer to the colony. However, Group 1 tended to stay within the 200 m bathymetry contour whereas Group 2 tended to forage in deeper waters (Fig. 1) and spent a lower proportion of time at sea diving (41.4 ± 4.1%).

Group 3 was comprised of all trips completed by individuals GSL5, GSL6, GSL7 and GSL9. This group used a combination of Dive Types with EP and DB being the most common (49.8 ± 9.4% and 37.1 ± 8.8%, respectively). These individuals completed on average more dives (437 ± 68) with greater dive durations (4.0 ± 0.5 min) and depths (126.9 ± 12.9 m) than individuals in Groups 1 and 2. Correspondingly, average post-dive duration (8.6 ± 2.6 min) was also greater in Group 3 than the other groups (1.8 ± 0.3 and 2.7 ± 0.6 min, respectively). Individuals from Group 3 tended to spend a longer time at sea (59.7 ± 6 h), with 43.7 ± 1.3% of this time spent diving. Spatial tracking showed individuals from this group utilised various environments including the continental shelf (0–200 m) and the continental slope (200–2,000 m), as well as the various islands used for hauling out. Individuals hauled out for 7.8 ± 3.8 h per trip at other islands, including Santa Fe Island and Santa Cruz Island to the west and Espanola Islands to the south (Fig. 1).

The results of the ANOVA on the LME indicated Foraging Trip Type had a significant influence on trip PPC (DF = 4, *F*_4_ = 14.38, *P* = 0.0149). Tukey’s *post-hoc* analysis determined Groups 1 and 2 were not significantly different (*P* = 0.986). However, Group 3 was significantly different from both Group 1 and 2 (*P* < 0.00001 in both cases). Groups 1 and 2 had a similar amount of average PPC per trip (123.5 ± 36.8 and 101.7 ± 27.8, respectively) and PPC rates per dive (0.6 ± 0.1 and 0.4 ± 0.1 respectively). For Group 1, PPC was predominant during SB dives whereas in Group 2, the majority of PPC was during EP dives. In comparison, Group 3 had a higher average number of PPC per trip (779.6 ± 190.5) and, therefore, a greater PPC rate (1.8 ± 0.4). The dives with the highest PPC rate in Group 3 were from DB (0.9 ± 0.2) and MP dives (0.5 ± 0.2), although PPC also occurred in EP dives at a lower rate (0.3 ± 0.1).

Foraging Trip Type was shown by the LME to influence both the energy consumed and expended (ANOVA:DF = 4, *F*_4_ = 13.99, *P* < 0.02 and DF = 4, *F*_4_ = 17.56, *P* < 0.01, respectively: Table S2). Energy consumption was greater than energy expenditure in all groups by a factor of approximately 10. Groups 1 and 2 were not significantly different in terms of the energy consumed or expended (Tukey’s: *P* > 0.5 in both cases: Table S3). However, Group 3 was significantly greater than Groups 1 and 2 for both energy consumed, and energy expended (*P* < 0.0001 in both cases). Consequently, there were no significant differences in foraging efficiency between the Foraging Trip Type groups (*F*_4_ = 2.14, *P* = 0.2333). In addition, no significant influence of individual body mass or morphometrics on Foraging Trip Type used could be detected (*P* > 0.1 in all cases).

## Discussion

Optimising energy gained relative to energy expended over a given time is the basic concept of foraging efficiency and is essential to an individual’s survival (MacArthur & Pianka, 1966; Pyke, 1984). Foraging efficiency can be influenced by factors such as predator avoidance, the environment, age and sex (Gallagher et al., 2015). While divergence of foraging strategies is considered to reduce competition (MacArthur & Pianka, 1966), little is known of the energetic consequences of their adaptations (Bolnick et al., 2003; Houston & McNamara, 2014). In the present study, while differences in the prey capture rates and energy expenditure in relation to the foraging strategy employed (at both the dive level and trip level) were found, the foraging efficiency of GSL was similar across strategies. These findings suggest that individuals modify foraging outcomes in order to adapt to various environmental, anthropogenic and ecological pressures.

### Potential prey captures, dive behaviour and foraging efficiency

In the present study, PPC were estimated utilising accelerometer data following methods previously validated with animal-borne video data loggers (88.3% precision) in another otariid species (Volpov et al., 2015; Foo et al., 2016). While it is possible that the number of PPC recorded in the present may have over- or underestimated prey captures, the average PPC rate of 0.97 per dive is comparable to that observed in Australian fur seals (Foo et al., 2016), Antarctic fur seals (Jeanniard-du Dot et al., 2017a), and northern fur seals (Jeanniard-du Dot et al., 2018).

The rate of PPC in the present study was strongly influenced by the Dive Type, with Benthic dives having a lower PPC rate than pelagic dives within the same depth range. Previous studies have suggested that individuals will have fewer prey encounters when feeding on larger, more energy-dense prey items, a trend observed in benthic foragers (Costa, 1991; Naito et al., 2013). However, in the present study a higher PPC rate was observed in both the deeper MP and DB dives. There is a potentially greater energetic cost associated with increased transit times in deep diving individuals (Rosen, Winship & Hoopes, 2007), which is possibly outweighed by a greater prey abundance and/or prey energetic content at those depths.

Sustainable dive strategies should have a favourable ratio of energy gain to cost (Emlen, 1966; MacArthur & Pianka, 1966). The GSL benthic dives had a higher energy expenditure rate than pelagic ones occurring to similar depths. This is consistent with recent studies of New Zealand sea lions (*Phocarctos hookeri*) where benthic foraging strategies appeared to be more energetically expensive than the other strategies observed in that species (Chilvers & Wilkinson, 2009). Benthic divers tend to spend a greater amount of time (and energy) foraging at depth and, therefore, must increase energy consumption by targeting larger prey or higher energy content items to maintain foraging optimality (Costa, 1991; Costa et al., 2004). Pelagic divers in the current study conducted dives of shorter duration than benthic divers when foraging at similar depths as has been observed in Californian sea lions (*Zalophus californianus*) (McHuron et al., 2016). Pelagic foragers also tend to consume smaller prey items, therefore, in order to gain adequate energy an individual must consume higher quantities of prey (Costa, 1991).

Mesopelagic dives were the exception to the positive ratio of energy consumed to energy expended in the present study. Since energy consumption was partially based on estimates of energy content of prey from previous studies, there may be biases in the estimations of prey energy content due to prey fluctuations between years or sampling bias. Nonetheless, MP dives are likely to have high energetic costs due to the high energetic cost of both transit and thermoregulation associated with achieving deeper dives. Páez-Rosas, Villegas-Amtmann & Costa (2017) reported the predominant mesopelagic species in the GSL diet to be Lanternfish (Myctophidae) and Panama lightfish (*Vinciguerria lucetia*). Both are small, deep pelagic fish of high energy content. Lanternfish, in particular have high levels of lipids including high levels of triglyceride amongst up to 14 other fatty acids in some species (Chai et al., 2012), potentially providing critical nutritional components to the GSL diet.

Previous studies have revealed the GSL diet is often dominated by 4–8 species of importance from distinct habitats that are complemented by a range of other species that make up a small proportion of their diet (Páez-Rosas & Aurioles-Gamboa, 2010; Páez-Rosas & Aurioles-Gamboa, 2014). Pelagic prey items such as Big-eye scad (*Selar crumenophthalmucs*) or Lanternfish are small vertically migrating fish with high energy density. In comparison, benthic prey items such as rock cod or groupers (Serranidae) and scorpion fish (Scorpaenidae) tend to be larger and have lower energy density (Eder & Lewis, 2005; Kumar et al., 2014; Páez-Rosas, Villegas-Amtmann & Costa, 2017). Although pelagic divers may have to invest more effort in terms of the number of PPC per dive, the energy density of their prey is generally higher, with the opposite being true for benthic divers.

### Influence of foraging trip behaviour on foraging efficiency

GSL are considered generalist foragers, although recent studies have observed individuals from various colonies specialising their diet and foraging methods (Villegas-Amtmann et al., 2008; Villegas-Amtmann et al., 2013; Páez-Rosas & Aurioles-Gamboa, 2010). This trend has also been observed in other generalist populations including pinnipeds such as the Californian sea lion (*Z. californianus*) (McHuron et al., 2016), New Zealand sea lions (Chilvers & Wilkinson, 2009), and northern fur seals (Jeanniard-du Dot et al., 2018), as well as in sea otters (Lee, Gelwick & Davis, 2010), and seabirds (Camprasse et al., 2017). The variation of foraging behaviours or niche separation of individuals within a population should result in reduced competition pressures and increase foraging efficiency of individuals (Van Valen, 1965).

In previous studies of niche segregation in GSL, age and mass were determined to have some influence on foraging behaviour (Jeglinski et al., 2013). Similar finding have also been observed in California sea lions (Weise, Harvey & Costa, 2010) and Gentoo penguins (*Pygoscelis papua*) (Camprasse et al., 2017). In contrast, no significant differences in morphometrics or body condition indices were observed between the different Foraging Trip Types in the present study. While this could be due to the low sample size and variation in the individuals sampled, similar findings have been reported for New Zealand sea lions (Chilvers & Wilkinson, 2009).

Three Foraging Trip Types were observed in the present study. Two of the foraging strategies were comprised of individuals that predominately utilised either shallow benthic or epipelagic dive strategies throughout the trip. These individuals tended to complete shorter trips and had lower PPC rates and ratios between energy consumed to energy expended. Individuals that utilised the remaining strategy had predominantly longer foraging trips and spent a greater amount of time diving. Consequently, these individuals expended significantly more energy within a foraging trip. This could be related to distance travelled and the energetic costs of thermoregulation due to greater time at sea (Montero-Serra et al., 2014). Similar studies have found that increased field metabolic rate and, therefore, energy expenditure, are higher in individuals that complete extended foraging periods (Villegas-Amtmann et al., 2017).

Despite variation in energy consumption, energy expenditure, and dive parameters between groups, foraging efficiency was not significantly different between groups. The mean foraging efficiency of Foraging Trip Types ranged from 0.4 to 1.3 kJ s^−1^, values consistent with estimates for Antarctic fur seals (2.02 kJ s^−1^) (Jeanniard-Du-dot et al., 2017b) and northern fur seals (∼0.15 kJ s^−1^) (Jeanniard-du Dot et al., 2018). Similar to GSL, the variation in foraging efficiency in these species are linked to the foraging behaviours. In northern fur seals, the individuals predominately using shallow benthic foraging were foraging closer to shore. They had lower foraging efficiency and PPC rates than individuals that foraged further from the colony and utilised predominantly pelagic foraging.

In 2012, the Galapagos region experienced a La Niña event, which is usually associated with cooler waters and greater productivity (NOAA, 2020). However, the 2012 La Niña was unusually warm (NOAA, 2020) and may have contributed the variation in GSL foraging trip strategies observed in the present study. Nonetheless, the variation in observed foraging trip strategies was not associated with differences in foraging efficiency. This suggests that the various currents, habitats and bathymetry surrounding the Galapagos Islands have enabled GSL to adapt various successful foraging behaviours (Drago et al., 2016; Páez-Rosas, Villegas-Amtmann & Costa, 2017). However, with the increasing frequency of major El Niño events that influence changes in climate, ecological interactions, and food availability (Cai et al., 2014), the flexibility of foraging behaviours in GSL and the ability to adapt to these stresses may be impacted (Trillmich et al., 2014). Given the current study covered only 1–7 foraging trips over a 1 month period and it is not known whether the strategies observed reflect individual specialisation (Kernaléguen et al., 2016). Further longitudinal studies are required to determine whether the observed differences in Foraging Trip Type reflect individual foraging specialisation and if they confer life-history advantages.

##  Supplemental Information

10.7717/peerj.11206/supp-1 Figure S1 Hierarchical cluster analysis of dive parameters depicting trips (*n* = 25) and individual Galapagos sea lions (*n* = 7) utilising similar Foraging Trip Types in November 2012 A 95% CI was used to determine the cut off based on the Euclidean distance with the ward.D2 linking method. Group 1 = red, Group 2 = blue, Group 3 = green.

10.7717/peerj.11206/supp-2 Table S1 Estimated energy content of important prey species in Galapagos sea lion diet Diet estimations based on Páez-Rosas, Villegas-Amtmann & Costa (2017). Energy content estimates (kJ) were taken from the literature and FishBase based on estimated prey mass (g) and length (cm).

10.7717/peerj.11206/supp-3 Table S2 ANOVA output run on Foraging Trip Type and Dive Type Linear Mixed Effect Models Fixed effects assessed include: Energy Consumed, Energy Expended, Foraging Efficiency and Potential Prey Captures displaying degrees of freedom (DF), F-value (F) and P-values (P).

10.7717/peerj.11206/supp-4 Table S3 Tukeys *post-hoc* analysis output from Linear Mixed Effect Models, analysis was run on Foraging and Dive Types Linear Mixed Effect Models assessing fixed effects: Energy Consumed, Energy Expended, Foraging Efficiency and Potential Prey Captures (PPC) in Foraging Types (Groups 1, 2 and 3) and Dive Types (deep benthic (DB), shallow benthic (SB), mesopelagic (MP), and epipelagic (EP)). All values are displayed as *P*-values.

10.7717/peerj.11206/supp-5 Data S1 GPS and ARGOS location data including latitude, longitude, time and date, and individual for sampled Galapagos sea lions (*n* = 8) from the El Malecon Colony

## Additional Information and Declarations

### Field Study Permissions

The following information was supplied relating to field study approvals (i.e., approving body and any reference numbers): This research has been carried out as part of a research project authorized by the Directorate of the Galapagos National Park (DPNG), Project name: PC-19-12 and PC-61-13. “Ecología trófica de los pinnípedos de las Islas Galápagos: Análisis regional y temporal” granted to Dr. Diego Páez-Rosas and respecting all the DPNG regulations.

### Data Availability

The following information was supplied regarding data availability: The location data, either ARGOS or GPS, is available in the Supplemental Files. IgorPro project files containing combined dive behaviour and accelerometer data for each individual are available at Zenodo: Blakeway, Jessica-Anne; Arnould, John PY; Hoskins, Andrew J; Martin-Cabrera, Patricia; Sutton, Grace J; Huckstadt, Luis A; Costa, Daniel P; Paez-Rosa, Diego; Villegas-Amtmann, Stella. (2020). Galapagos sea lion foraging success [Data set]. Zenodo. http://doi.org/10.5281/zenodo.4059111.
