# Supplementary figures and images for: Influence of hunting strategy on foraging efficiency in Galapagos sea lions
Source: PeerJ. 2021 Apr 13;9:e11206. doi: 10.7717/peerj.11206 (PMC8051337; doi:10.7717/peerj.11206)

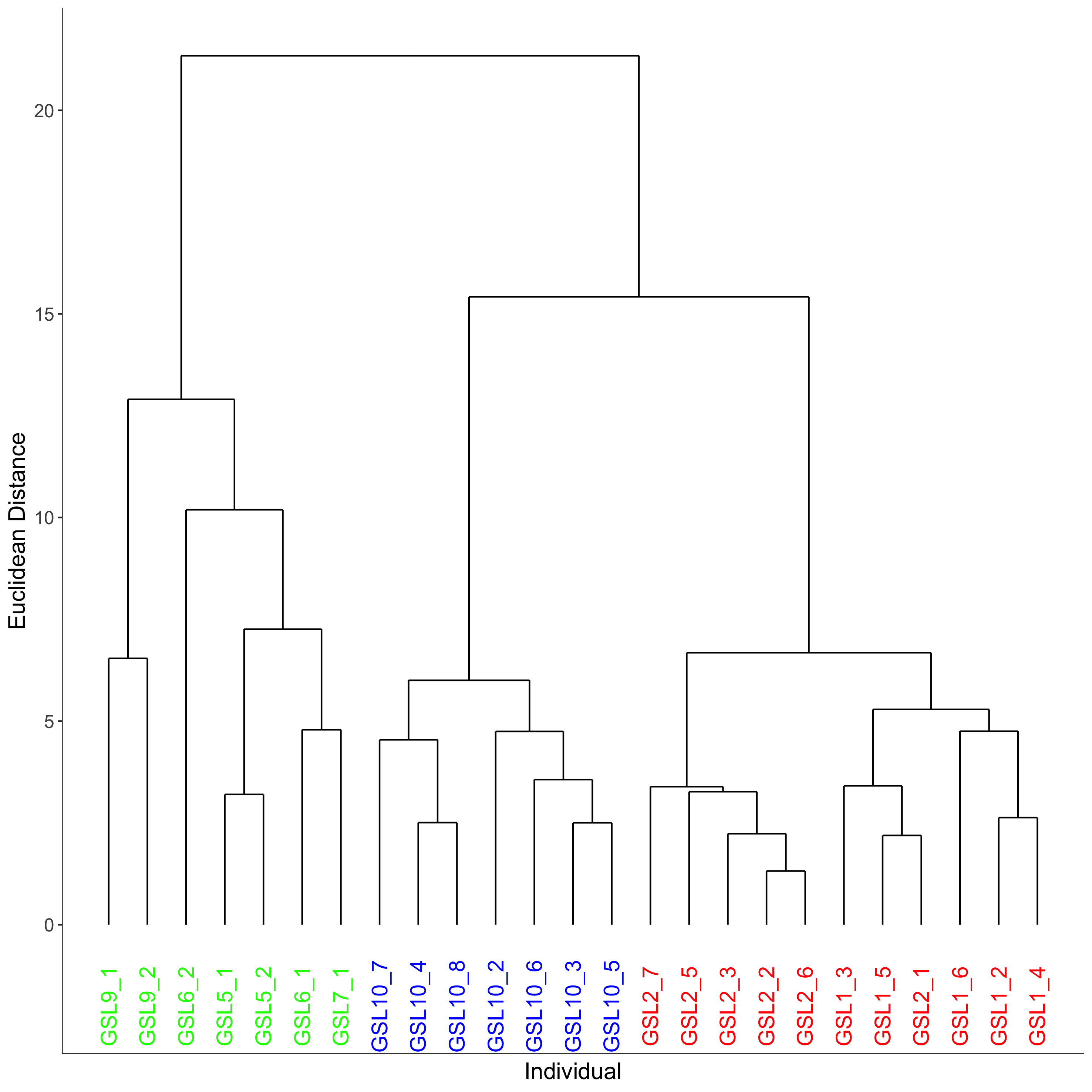

Supplement: Figure S1 — A 95% CI was used to determine the cut off based on the Euclidean distance with the ward.D2 linking method. Group 1 = red, Group 2 = blue, Group 3 = green. [file peerj-09-11206-s001.png]
