# Supplementary material for: Influence of hunting strategy on foraging efficiency in Galapagos sea lions
Source: PeerJ. 2021 Apr 13;9:e11206. doi: 10.7717/peerj.11206 (PMC8051337; doi:10.7717/peerj.11206)
Supplement: Table S1 — Diet estimations based on Páez-Rosas, Villegas-Amtmann & Costa (2017). Energy content estimates (kJ) were taken from the literature and FishBase based on estimated prey mass (g) and length (cm). [file peerj-09-11206-s002.docx]

| Dive Type | Scientific name | Common name | Mass  (g) | Length  (cm) | Average Gross Energy Content (kJ) |
| --- | --- | --- | --- | --- | --- |
| Pelagic | *Selar crumenophthalmus* | Big eyed scad | 54.5^1^ | 20^1^ | 380.20^1^ |
|  | *Opisthonema berlanga* | Galapagos sardine | 68.74^1^ | 18^1^ |  |
| Mesopelagic | *Myctophidae spp.* | Lanternfishes | 1.45^1^ | 6.5^1^ | 9.51^1^ |
|  | *Vinciguerria lucetia* | Panama lightfish | 0.65^1^ | 4.5^1^ |  |
| Shallow Benthic | *Serranidae spp.* | Rock cod & groupers | 225^2^ | 22^2^ | 801.41^2,3,4^ |
|  | *Synodus lacertinus* | Sauro lizardfish | 72.5^2^ | 20.5^2^ |  |
|  | *Otophidium indefatigable* | Brownspot cusk eel | 12.5^1^ | 12^1^ |  |
|  | *Pontinus clemensi* | Mottled Scorpionfish | 333^4^ | 25^4^ |  |
| Deep Benthic | *Physiculus nematopus* | Charcoal Codling | 17.8^4^ | 38.9^4^ | 679.15^2,4^ |
|  | *Serranidae spp.* | Rock cod & groupers | 225^2^ | 22^2^ |  |

References for mass, length and gross energy content estimates:

^1^(Froese & Pauly, 2019)

^2^(Kumar *et al.*, 2014)

^3^ (Crabtree, 1995)

^4^ (Eder & Lewis, 2005)
