# Supplementary material for: Influence of hunting strategy on foraging efficiency in Galapagos sea lions
Source: PeerJ. 2021 Apr 13;9:e11206. doi: 10.7717/peerj.11206 (PMC8051337; doi:10.7717/peerj.11206)
Supplement: Table S2 — Fixed effects assessed include: Energy Consumed, Energy Expended, Foraging Efficiency and Potential Prey Captures displaying degrees of freedom (DF), F-value (F) and P-values (P). [file peerj-09-11206-s003.docx]

| ANOVA Output | | DF | F | P |
| --- | --- | --- | --- | --- |
| Foraging Type | Energy Consumed | 18 | 45.157 | <0.0001 |
|  | Energy Expended | 18 | 255.208 | <0.0001 |
|  | Foraging Efficiency | 18 | 42.495 | <0.0001 |
|  | Potential Prey Capture | 18 | 29.249 | <0.0001 |
|  |  |  |  |  |
| Dive Type | Energy Consumed | 10249 | 46.837 | <0.0001 |
|  | Energy Expended | 10249 | 47.395 | <0.0001 |
|  | Foraging Efficiency | 10249 | 66.286 | <0.0001 |
|  | Potential Prey Capture | 10249 | 64.471 | <0.0001 |
