# Supplementary material for: Influence of hunting strategy on foraging efficiency in Galapagos sea lions
Source: PeerJ. 2021 Apr 13;9:e11206. doi: 10.7717/peerj.11206 (PMC8051337; doi:10.7717/peerj.11206)
Supplement: Table S3 — Linear Mixed Effect Models assessing fixed effects: Energy Consumed, Energy Expended, Foraging Efficiency and Potential Prey Captures (PPC) in Foraging Types (Groups 1, 2 and 3) and Dive Types (deep benthic (DB), shallow benthic (SB), mesopelagic (MP), and epipelagic (EP)). All values are displayed as P-values. [file peerj-09-11206-s004.docx]

| Group Comparisons | | Gross Energy Consumed | Gross Energy Expended | Foraging Efficiency | PPC |
| --- | --- | --- | --- | --- | --- |
| 1 | 2 | 0.5060 | 0.8820 | 0.1610 | 0.9860 |
| 1 | 3 | 0.0001 | 0.0000 | 0.9960 | 0.0000 |
| 2 | 3 | 0.0001 | 0.0000 | 0.1240 | 0.0000 |
|  |  |  |  |  |  |
| DB | SB | 0.0000 | 0.0010 | 0.2530 | 0.0001 |
| EP | SB | 0.0000 | 0.0010 | 0.0001 | 0.3360 |
| MP | SB | 0.0000 | 0.0010 | 0.0001 | 0.0001 |
| EP | DB | 0.0000 | 0.0010 | 0.0001 | 0.0001 |
| MP | DB | 0.0000 | 0.0414 | 0.0001 | 0.0001 |
| MP | EP | 0.0000 | 0.0010 | 0.0001 | 0.0001 |
